# Potential use of the *Eucalyptus globulus* vacuolar pyrophosphatase 1 (EgEVP1) to generate drought and salt resistant plants

**DOI:** 10.1186/1753-6561-5-S7-O34

**Published:** 2011-09-13

**Authors:** Erwin Krauskopf, Maria Cecilia Gamboa

**Affiliations:** 1 Facultad de Ciencias Biologicas, Universidad Andres Bello, Santiago, Chile; 2 Fundacion Ciencia para la Vida, Santiago, Chile

## 

Desertification is a phenomenon that affects vast territories worldwide usually caused by high soil salt concentrations, being salt and water stress one of the main problems that affect plant growth. Part of the strategy developed by the plant to face these stresses consists on the compartmentalization of cytoplasmic Na^+^ within the vacuole, by a coordinated expression of a H^+^ pump that uses pyrophosphate as a source of energy (known as vacuolar pyrophosphatase) and a Na^+^/H^+^ exchanger, both located on the vacuole membrane. The vacuolar pyrophosphatase has been described in various plant species, much of which are of commercial importance, such as rice, wheat and corn, along some woody species such as poplar and apple. In our laboratory we isolated the cDNA of the *Eucalyptus globulus* vacuolar pyrophosphatase (EVP1), establishing by real-time PCR that its transcript expression increases when *Eucalyptus globulus* plants are subjected to water and salt stress. An analysis of its genomic sequence allowed us to identify four introns and a promoter region of approximately 2Kb. The fusion of the GUS reporter gene to the promoter region as well as to the first EVP1 intron allowed us to establish for the latter a possible regulatory function, in addition to identifying the promoter tissue-specific expression in *A.thaliana* plants. Finally, *A. thaliana* transgenic plants generated by overexpressing EVP1 produced seeds that exhibited a higher germination rate in high salt conditions compared to the wild-type plants. Furthermore, adult transgenic plants had more leaves in the rosette and a higher tolerance to salt and water stress.

Funded by: PFB-016 and DI-UNAB

